# Estradiol deficiency reduces the satellite cell pool by impairing cell cycle progression

**DOI:** 10.1152/ajpcell.00429.2021

**Published:** 2022-04-20

**Authors:** Alexie A. Larson, Ahmed S. Shams, Shawna L. McMillin, Brian P. Sullivan, Cha Vue, Zachery A. Roloff, Eric Batchelor, Michael Kyba, Dawn A. Lowe

**Affiliations:** ^1^Department of Integrative Biology and Physiology, Medical School, University of Minnesota, Minneapolis, Minnesota; ^2^Lillehei Heart Institute, Medical School, University of Minnesota, Minneapolis, Minnesota; ^3^Department of Pediatrics, Medical School, University of Minnesota, Minneapolis, Minnesota; ^4^Human Anatomy and Embryology Department, Faculty of Medicine, Suez Canal University, Ismailia, Egypt; ^5^Divisions of Rehabilitation Science and Physical Therapy, Department of Rehabilitation Medicine, Medical School, University of Minnesota, Minneapolis, Minnesota

**Keywords:** muscle stem cells, ovariectomy, satellite cell cycling, skeletal muscle

## Abstract

The size of the satellite cell pool is reduced in estradiol (E_2_)-deficient female mice and humans. Here, we use a combination of in vivo and in vitro approaches to identify mechanisms, whereby E_2_ deficiency impairs satellite cell maintenance. By measuring satellite cell numbers in mice at several early time points postovariectomy (Ovx), we determine that satellite cell numbers decline by 33% between 10 and 14 days post-Ovx in tibialis anterior and gastrocnemius muscles. At 14 days post-Ovx, we demonstrate that satellite cells have a reduced propensity to transition from G_0_/G_1_ to S and G_2_/M phases, compared with cells from ovary-intact mice, associated with changes in two key satellite cell cycle regulators, *ccna2* and *p16^INK4a^*. Further, freshly isolated satellite cells treated with E_2_ in vitro have 62% greater cell proliferation and require less time to complete the first division. Using clonal and differentiation assays, we measured 69% larger satellite cell colonies and enhanced satellite cell-derived myoblast differentiation with E_2_ treatment compared with vehicle-treated cells. Together, these results identify a novel mechanism for preservation of the satellite cell pool by E_2_ via promotion of satellite cell cycling.

## INTRODUCTION

Skeletal muscle growth and regeneration require a mitotically quiescent stem cell population known as satellite cells (1, 2). During homeostasis, satellite cells reside on the periphery of terminally differentiated muscle fibers and are marked by the expression of paired box transcription factor 7 (Pax7) and several cell surface markers, including α7-integrin (3–6). Following a stimulus (e.g., injury or stress), a subset of the satellite cells transition from a quiescent to activated state and enter the G_1_ phase of the cell cycle (7). Satellite cells can then undergo asymmetric division where one daughter cell commits to the myogenic lineage, differentiates and fuses to new or existing damaged fibers, and the other daughter cell returns to quiescence to maintain the satellite cell pool, a process known as self-renewal (8). Satellite cells can also undergo symmetric proliferation followed by stochastic recruitment of proliferated progeny back into the satellite cell pool (9, 10). In healthy adult muscle, the appropriate balance of intrinsic and extrinsic factors is maintained to coordinate satellite cell fate decisions (i.e., myogenic commitment vs. self-renewal) with the demands of regenerating or growing muscle (11, 12).

The effects of disrupting the complex balance of factors that affect satellite cell fate can be observed in aging skeletal muscle, resulting in markedly compromised muscle regeneration. Major advances toward understanding how changes in the intrinsic and extrinsic factors that influence the satellite cell pool have been made in the last 60 yr since the satellite cell was discovered (13). Numerous groups have observed a decline in satellite cell number in aged rodents (14–22) and human skeletal muscles (23–25) with the rate and extent varying with muscle fiber type and function (e.g., locomotion, respiration, or mastication; 18, 20, 21). In addition, several studies have demonstrated that changes in extrinsic factors in the satellite cell microenvironment contribute to impaired regeneration with age (reviewed in Refs. 24–30). The decline of circulating hormones including insulin-like growth factor-1 (IGF-1) and oxytocin have been identified as contributors to age-associated impairments of satellite cells (31–33). Over the last decade, the relationship between satellite cell function and sex hormones has gained attention to rationalize sex-related differences in skeletal muscle regeneration (34–43). In particular, evidence is mounting that the major sex hormone in females, estradiol (E_2_), influences satellite cell function and muscle regeneration.

It is important to understand that E_2_ levels can decline in females due to a variety of reasons, including *1*) natural age-induced menopause (44), *2*) menstrual dysfunction experienced with the female athlete triad (45), *3*) side effects of hormone therapy to treat cancer (46), *4*) congenital conditions (e.g., Turner syndrome; 47, 48), and *5*) hysterectomy with or without oophorectomy (i.e., surgical removal of the ovaries; referred to as ovariectomy in animals; 49). Health issues associated with E_2_ deficiency traditionally prompted studies focused on osteoporosis (reviewed in Ref. 50) and heart disease (reviewed in Ref. 51) leaving the role of E_2_ on skeletal muscle and satellite cell biology less clear. Early studies have shown that E_2_ deficiency in females blunts satellite cell activation and proliferation induced by injury or exercise (51–56). Interestingly, E_2_ treatment has been shown to both impair and enhance satellite cell differentiation in mice (C2C12) and rat (L6) myoblast cells (57–59). Inconsistencies in the effects of E_2_ on satellite cells are presumably due to different experimental conditions including animal model and age, E_2_ dose and treatment duration, as well as methods of measuring progression of satellite cells through myogenesis. Although these studies suggest potential mechanisms of action of E_2_ on muscle regeneration, well-defined mechanisms, whereby E_2_ regulates satellite cell function are yet to be determined.

Our recent findings show that the size of the satellite cell pool is reduced in ovarian hormone-deficient female mice and humans under normal homeostatic conditions (60, 61). Using hormone replacement, we demonstrate that E_2_ is the ovarian hormone responsible for affecting satellite cells (60, 61). Here we investigate mechanisms whereby the loss of circulating E_2_ in females results in the reduced satellite cell number under normal homeostatic conditions, i.e., without any muscle injury. We posit that E_2_ influences satellite cell maintenance by regulating satellite cell cycle kinetics, progression, proliferation, and differentiation. To test this hypothesis, we use ovariectomized (Ovx) female mice to study satellite cell biology in vivo and thus examine the cell cycle progression of satellite cells with and without circulating E_2_. In addition, considering that satellite cells are heterogeneous regarding their cell cycle progression, we assess cell cycle kinetics, proliferation, and differentiation in vitro by treating freshly isolated satellite cells from female mice with E_2_. Our results show decrements in satellite cell cycle progression with E_2_ deficiency, suggesting that satellite cell number declines due to inability of the satellite cells to cycle and generate progeny without E_2_ in the environment. These findings have implications in the preservation of efficient muscle regeneration, including targeting p16-mediated pathways to prevent cell cycle arrest of satellite cells and subsequent exhaustion of the satellite cell pool. Ultimately, understanding how E_2_ regulates satellite cells will help to determine therapies for improving muscle regeneration and recovery of strength that affect the quality of life.

## METHODS

### Mice

All procedures were performed in accordance with protocols approved by the Institutional Animal Care and Use Committees at the University of Minnesota (No. A3456-01). All experiments were conducted on female mice when they were mature adults (3–6 mo of age, life phase equivalent of ∼20–30 yr for humans; 62). Female wild-type (C57Bl/6) mice were obtained from Jackson Laboratory (000664; Bar Harbor, ME). Female Pax7-ZsGreen, *Pax7^CreERT2/+^; Esr1^fl/fl^;Pax7-ZsGreen* (scERαKO), and *Pax7^+/+^Esr1^fl/fl^;Pax7-ZsGreen* (scERαWT) mice were generated in-house (4). Mice were housed in groups of 4–5 and had access to phytoestrogen-free rodent chow (Harlan-Tekland No. 2019; Indianapolis, IN) and water ad libitum. The housing room was maintained on a 14:10 light:dark cycle with controlled temperature and humidity.

For time-course experiments, female C57Bl/6 mice were assigned to one of two groups: Sham or Ovariectomized (Ovx) and were euthanized 6, 10, or 14 days postsurgery (*n* = 25 for each group). Female scERαWT and scERαKO mice were treated with tamoxifen (2 mg/kg) for 5 days consecutively (63, 64). At 14 days after tamoxifen treatment, mice were used for satellite cell harvests (*n* = 6/group). For the *Pax7^CreERT2/+^* effect experiment, female scERαWT and scERαKO mice were used for satellite cell harvests (*n* = 6/group). For the tamoxifen effect experiment, female scERαWT were treated with a vehicle [15% ethanol (EtOH) in sunflower seed oil] or tamoxifen (*n* = 5/group). For in vivo proliferation experiments, female C57Bl/6 mice that were either Sham or Ovx received 21 days slow-release 5-ethynyl-2′-deoxyuridine (EdU; 25 mg) immediately following surgery (*n* = 7–9). For in vitro experiments, satellite cells were harvested from Pax7-ZsGreen female mice (*n* = 3–6/group). Mice were euthanized with an intraperitoneal injection of sodium pentobarbital (200 mg/kg) followed with cervical dislocation as secondary euthanasia.

### Surgical Procedures

Sham and Ovx surgeries were performed as previously described (65). Briefly, mice were given a subcutaneous injection of slow-release buprenorphine (1 mg/kg), and 2–4 h later were anesthetized by inhalation of isoflurane (2%–3%, 125 mL O_2_/min). Bilateral Ovx was performed through two small dorsal incisions between the iliac crest and the lower ribs, and Sham operations consisted of the same procedure as Ovx except that the ovaries were not removed. In a subset of mice, immediately after Ovx, mice were implanted with pellets containing 25 mg EdU released over a 21 days period (Innovative Research of America, Sarasota, FL). The daily dose of EdU is equivalent to that given by intraperitoneal injection daily (50 mg/kg). Mice were monitored daily for 3 days following surgery, and incision wound clips were removed at 10 days postsurgery. The estrous cycle of Sham and Ovx mice was tracked for 3–5 days consecutively via vaginal cytology to confirm normal estrous cycles or persistent diestrus, respectively (66). At the completion of all experiments, uteri were dissected and weighed. Uterine mass <30 mg was used as an inclusion parameter to indicate successful Ovx surgery (67).

### Satellite Cell Isolation

Isolation of satellite cells from individual muscles [e.g., tibialis anterior (TA) and gastrocnemius (GC)] and bulk muscles (hindlimb muscles) were performed as described in detail previously (18, 60). Briefly, muscles were dissected, minced in parallel with muscle fibers, and digested with collagenase type II and dispase (17101-015 and 17105-041, respectively; Gibco, Grand Island, NY). Mononuclear cells were stained using an antibody mixture of 1 µL PE-Cy7 rat anti-mouse CD31 (clone 390; 561410; BD Biosciences, San Diego, CA), 1 µL PE-Cy7 rat anti-mouse CD45 (clone 30-F11; 552848; BD Biosciences), 1 µL biotin rat anti-mouse CD106 [clone 429 (MVCAM.A); 553331; BD Biosciences], 1 µL PE streptavidin (554061; BD Biosciences), and 2 µL α7 integrin 647 (clone R2F2; AbLab; Vancouver, BC, Canada). Samples were incubated with an antibody cocktail, washed, and resuspended with fluorescence-activated cell sorting (FACS) staining medium [2% fetal bovine serum (FBS; 16000044; Gibco) in phosphate-buffered saline (PBS)] containing 0.5 µg/mL propidium iodide (PI) for analysis on a FACSAriaII SORP (BD Biosciences, San Diego, CA). Total satellite cells (lineage negative; VCAM, α7 double-positive cells) were analyzed from the entire muscle sample. For isolation of satellite cells from Pax7-ZsGreen mice, mononuclear cells were incubated in FACS staining medium containing PI and ZsGreen+ cells were examined as described previously (68). Absolute satellite cell counts by FACS were confirmed through gating ZsGreen+ cells and counting beads (CountBright absolute counting beads; C36950; Lot No. 2361079; Invitrogen, Waltham, MA) according to the manufacturer’s instructions. Cell concentration was calculated using the formula: (number of cell events ÷ number of bead events) × (assigned bead count of the lot ÷ volume of sample).

### Pax7 Immunostaining

TA muscles were removed and placed in OCT compound, frozen in 2-methylbutane (Sigma-Aldrich), cooled by liquid nitrogen, and stored at −80°C until use. For visualization of satellite cells, Pax7 and laminin staining were performed on 7 µM cryosections (CM 1850, Leica Microsystems, Buffalo Grove, IL). Sections were fixed in 4% paraformaldehyde (PFA), washed with PBS, and boiled in heat-induced antigen retrieval buffer (1.8 mM citric acid and 8.2 mM sodium citrate in water) for 30 min using an Instant Pot pressure cooker (Instant Appliances). Sections were incubated for 10 min in H_2_O_2_ to block endogenous peroxidase activity and then blocked for nonspecific binding in 0.5% PerkinElmer TNB blocking reagent [0.1 M Tris-HCl, pH 7.5; 0.15 M NaCl; 0.5% tyramide signal amplification (TSA) blocking reagent, FP1020] for 1 h at room temperature. Following blocking, sections were incubated with anti-pax7 mouse IgG1 primary antibody (PAX7, Developmental Studies Hybridoma Bank, 1:10) and anti-laminin rabbit (L9393; Sigma-Aldrich, 1:250) in TNB blocking buffer overnight at 4°C. After washing with PBS, sections were incubated with goat anti-mouse biotin-conjugated secondary antibody (115-065-205; Jackson Immuno Research Laboratories Inc, West Grove, PA; 1:1,000) and Alexa Fluor 488 goat anti-rabbit (A11034; Invitrogen; 1:500) in TNB blocking buffer for 2 h at room temperature. Visualization of the Pax7 primary antibody was achieved by incubating the sections with the Vectastain ABC reagent (PK-6100; Vector Laboratories, Burlingame, CA) for 3 h and incubation in the dark with TSA cyanine 3 kit (NEL744; PerkinElmer, Waltham, MA; 1:50) in diluent buffer for 10 min. Finally, the sections were mounted with antifade Prolong gold with 4,6-diamidino-2-phenylindole (DAPI). All images were processed and analyzed in a blinded manner with samples being de-identified as to the group. Mouse muscle samples were examined and imaged using a Leica DM5500B microscope (Leica Microsystems) at ×5 to ×20 magnification. Images were stitched using the automated tile-scan tool to construct an image of the entire cross section of the TA muscle. Satellite cells were identified by DAPI^+^ and Pax7^+^ cells residing along the myofiber border and were quantified using the region of interest (ROI) manager in the ImageJ software package (NIH, Bethesda, MD). For determination of the cross-sectional area of the TA muscle, the freehand and wand selection tools of the ImageJ were used to measure maximum Feret’s diameter.

### DNA Content Analysis

Pax7-ZsGreen cells were isolated by FACS and fixed by adding cooled 70% EtOH dropwise while vortexing cell suspension. Cells were then washed with PBS and incubated in a staining solution containing 0.1% (vol/vol) Triton-X100 in PBS, 2 mg DNase-free RNase (Sigma), and 1 mg/mL PI for 30 min at 37°C. Samples were analyzed on a FACSAriaII SORP (BD Biosciences, San Diego, CA). Cell cycle distributions for satellite cells in G_1_, S, and G_2_ phases were performed using FlowJo v.10 univariate modeling with the Watson pragmatic algorithm.

### RT-qPCR

RNA from freshly FACS-isolated satellite cells was isolated using Qiagen RNeasy Plus Universal Mini kit (73404; Hilden, Germany) according to manufacturer’s instructions. cDNA was synthesized from 100 ng RNA according to directions in SUPERVILO cDNA Synthesis Kit (11756050; Thermo Fisher Scientific, Waltham, MA). Relative quantitation of *cdkn1b/p27^Kip1^* (Mm00438168_m1), *cdkn2a/p16^INK4a^* (Mm00494449_m1), *ccnd1* (Mm00432359_m1), *ccna2* (Mm00438063_m1), *mapk14/p38* (Mm01301009_m1), and house-keeping gene *GAPDH* (Mm99999915_g1) were determined using TaqMan fast advanced master mix (4444557; Thermo Fisher Scientific; Waltham, MA).

### In Vivo EdU Proliferation Assay

Sham and Ovx mice received a pellet containing 25 mg EdU. Following 21 days of exposure, flow cytometry analysis was performed as described in the Click-iT EdU Alexa Fluor 488 Flow Cytometry kit (C104020; Invitrogen) combined with the mononuclear antibody mixture as described earlier in the satellite cell isolation section. A total of 50,000–100,000 events were recorded for the analysis. Proliferating satellite cells (i.e., S-phase satellite cells) were identified as lineage negative; VCAM, α7; FITC triple-positive cells (Supplemental Fig. S3*B*; see https://doi.org/10.6084/m9.figshare.17096918.v1). A positive control comprised of a 72 h post-barium chloride injured TA muscle was included to demonstrate robust EdU^+^ incorporation by satellite cells (Supplemental Fig. S3, *C* and *D*).

### In Vitro EdU Proliferation Assay

Pax7-ZsGreen cells were isolated by FACS and plated into 0.1% gelatin-coated 96-well plates (1,000 cells/well) containing muscle growth medium (MGM) with 20% charcoal-stripped (CS) FBS (NB036790; Thermo Fisher Scientific). The cells received MGM with or without E_2_ daily (100 pM final concentration; E8875; Sigma-Aldrich). On *day 6*, Click-iT EdU cell proliferation kit for imaging, Alexa Fluor 594 dye (C10339; Invitrogen) was performed according to the manufacturer’s instructions. The cells were then incubated in DAPI (1:1,000 dilution) in PBS for 20 min at room temperature. EdU+ nuclei were identified and imaged at ×10 magnification, taken on a Zeiss Observer.Z1 inverted microscope equipped with an AxioCam MRm camera (Thornwood, NY).

### MTT Proliferation Assay

Pax7-ZsGreen cells were isolated by FACS and plated into 0.1% gelatin-coated 96-well plate (2,000 cells/well) with Hams/F10 medium (SH30025.01; Hyclone, Logan, UT) supplemented with 20% CS-FBS (NB036790; Thermo Fisher Scientific), 10 ng/mL human basic fibroblast growth factor (bFGF; 100-18C; Peprotech), 1% Pen/Strep (15140122; Gibco), and 1% Glutamax (35050061; Gibco) and were incubated at 37°C and 5% CO_2_. Satellite cells were treated with E_2_ every 12 or 24 h to establish a final concentration of: 0 pM, 3.125 pM (0.85 pg/mL), 50 pM (13.62 pg/mL), or 100 pM (27.24 pg/mL) E_2_. The MTT assay was performed according to manufacturer’s instructions (11465007001; Roche). After 24 or 72 h, the MTT labeling reagent [3-(4,5-dimethylthiazol-2-yl)-2,5-diphenyltetrazolium bromide in PBS] was added to each well (final concentration 0.5 mg/mL) and incubated for 4 h. The solubilization solution was then added to each well and incubated overnight. The formazan product was measured in a microplate reader at a 570-nm wavelength.

### ATP Cell Proliferation Luciferase Assay

Pax7-ZsGreen cells were isolated by FACS and plated into 0.1% gelatin-coated 96-well plates (1,000 cells/well) containing MGM with 20% CS-FBS. The cells either received MGM with or without 100 pM E_2_. On *days 4* and *6*, CellTiter-Glo luminescent cell viability assay (G7570; Promega, Madison, WI) was performed. Medium was replaced with CellTitre-Glo reagent (1:3) in 100 µL of PBS. Plates were allowed to equilibrate for 3 min, then read on a Cytation3 plate reader (BioTek, Winooski, VT).

### Time to First Division and Cell Size

Pax7-ZsGreen cells were isolated by FACS and plated for live-cell imaging into 0.1% gelatin-coated 24-well glass-bottom dishes (NC9988706; Mattek; Thermo Fisher Scientific; Waltham, MA; 12,000 cells/well) containing MGM with 20% CS-FBS. Cells were treated with MGM containing vehicle (0.03% ethanol in PBS) or E_2_ (final concentration 100 pM) at the time of plating and again 18 h after plating. Time-lapse imaging was performed from 18 h to 72 h after plating with a Nikon Eclipse Ti-inverted fluorescence microscope equipped with an automated stage (Prior), and a custom chamber to maintain a constant 37°C temperature, high humidity, and 5% CO_2_. Multiple positions were analyzed per group with images acquired every 10 min using phase contrast. Images were collected using a ×20 CFI Plan Apochromat Lambda (NA = 0.75) objective (Nikon). For each condition, at least 100 individual cells were tracked. Following imaging, data were exported as individual TIFFs for each position and time point. ImageJ software package was used to concatenate TIFF images from each location and manually measure time to first division of each cell. Cell size at 18 h after plating was measured following pixel-based classification and cell segmentation with ilastik (version 1.3.3) and CellProfiler (version 4.0.5), respectively.

### Colony-Forming Assay

Pax7-ZsGreen cells were isolated by FACS and single cells were sorted into 0.1% gelatin-coated 96-well plates containing mouse myoblast medium (MMM): Dulbecco’s modified Eagle’s medium (DMEM; SH30284.01; Hyclone) without phenol red containing 4.00 mM l-glutamine, 4,500 mg/L glucose, and sodium pyruvate; 20% CS-FBS; 10% charcoal-stripped horse serum (CS-HS; NC9058780; Thermo Fisher Scientific); 10 ng/mL human basic fibroblast growth factor (bFGF; 100-18C; Peprotech), 1% Pen/Strep, and 1% Glutamax with or without E_2_ (final concentration 100 pM E_2_). Cells were allowed to adhere for 24 h and were then supplemented daily with MMM with or without 100 pM E_2_. After culturing plates for 8 days at 37°C and 5% CO_2_, cells were fixed with 4% PFA for 20 min at room temperature. For immunostaining of colonies, cells were permeabilized with 0.3% Triton-X100 for 20 min at room temperature, washed with PBS, and blocked with 3% BSA in PBS for 1 h at room temperature. Colonies were stained for MF-20 antibody supernatant (Developmental Studies Hybridoma Bank, University of Iowa; 1:20 dilution) in 3% BSA in PBS overnight at 4°C. After PBS washes, cells were incubated with Alexa Fluor 555 goat anti-mouse secondary antibody (Life Technologies; 1:500 dilution) in the dark for 45 min at room temperature. The cells were then incubated in DAPI (1:1,000 dilution) in PBS for 20 min at room temperature. Colonies were imaged at ×10 magnification, taken on a Zeiss Observer.Z1 inverted microscope equipped with an AxioCam MRm camera (Thornwood, NY). Intensity thresholding of ImageJ software package was used to measure the number of nuclei and number of colonies. The percentage of clonal efficiency was calculated by dividing the number of colonies in each plate by the number of wells in which a single cell was sorted then multiplying by 100. Colony size was measured using the freehand selection tool.

### Satellite Cell-Derived Myoblast Differentiation

Pax7-ZsGreen cells were isolated by FACS and plated into 0.1% gelatin-coated or Matrigel matrix (353234; Corning; Bedford, MA) 48-well plates (20,000 cells/well) containing MGM with 20% FBS. Cells were incubated at 37°C and 5% CO_2_ with MGM medium changed every other day. Cells reached 80%–100% confluence on *day 3* and were induced to differentiate in a low serum medium: DMEM supplemented with 2% normal or CS-HS, 1% Pen/Strep, and 1% Glutamax for 3.5 days with or without 100 pM E_2_. Immunofluorescent staining of cells for MF-20 and DAPI was performed as described in the clonal ability assay section. Fusion index was calculated as the percentage of nuclei in myotubes.

### Statistical Analysis

Two-way analysis of variance (ANOVA) was utilized to determine differences among times and groups. Holm–Sidak post hoc tests were performed in the event of a significant interaction or main effect of time. All other data were analyzed with two-tailed unpaired Student *t* tests for determining significant differences between two groups or one-way ANOVA with Holm–Sidak post hoc for determining significant differences among three or more groups. An α level of <0.05 was used for all analyses. Data are presented as means ± SE unless otherwise indicated. Time to first division data are presented as histograms representing individual cells dividing within time points and as scatter plots with means ± SD. Satellite cell size and colony size data are presented as scatter plots with means ± SD. All statistical testing was performed using GraphPad Prism 8.0 (GraphPad Software Inc., San Diego, CA) or SigmaPlot version 12.5 (Systat Software, San Jose, CA). Sample sizes are reported as the number of independent mice from which the cells were analyzed or isolated. All immunofluorescent images were processed and analyzed in a blinded manner with samples being de-identified as to treatment or group.

## RESULTS

**Figure 1.**
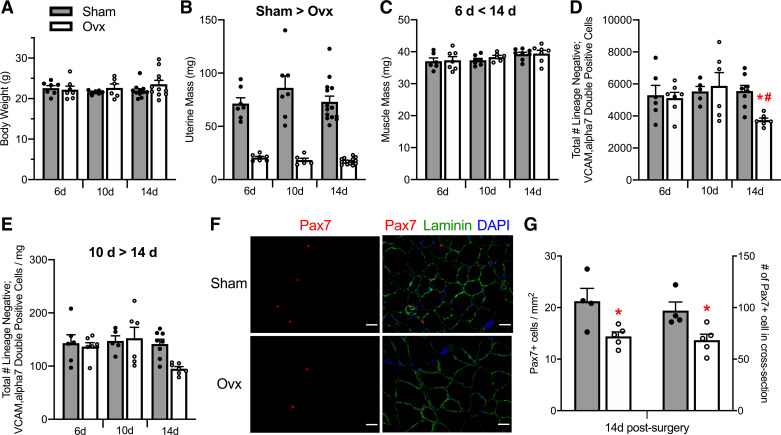
Effects of ovarian hormone deficiency on organ masses and satellite cell number. Body masses (*A)*, uterine masses *(B)*, and TA muscle masses of Sham and Ovx female mice (*n* = 6–14/group; *C*). *D*: total number of satellite cells quantified by FACS as lineage negative; VCAM, α7 double-positive cells in TA muscles 6, 10, or 14 days following a Sham or Ovx surgery. *E*: density of satellite cells as calculated from the total number of satellite cells normalized to wet TA muscle masses. *F*: satellite cell number quantified by Pax7 immunohistochemistry in TA muscle cross sections from Sham (*n* = 4) and Ovx (*n* = 5) at 14 days postsurgery. Pax7^+^ cells are presented normalized to TA cross-section area and as absolute satellite cells per cross section. Scale bars = 50 µm. Values are presented as means ± SE. Significant main effects of two-way ANOVAs (*P* < 0.05) are indicated above the bars (*B*, *C*, and *E*) and when significant interactions occurred, Holm–Sidak post hoc results are indicated by *different from Sham at corresponding time (*D*) and #different from 10 days Ovx (*D*). *Different from Sham by student *t* tests (*G*). FACS, fluorescence-activated cell sorting; Ovx; ovariectomized; TA; tibialis anterior.

Body mass did not differ between Sham and Ovx mice or across 6, 10, and 14 days postsurgery (*P* ≥ 0.416; Fig. 1*A*). Vaginal cytology confirmed estrous cycling in Sham mice and persistent diestrus in Ovx mice. Further, Ovx surgery was considered successful with uterine mass approximately, about fourfold less in Ovx than in Sham mice (*P* < 0.001) and all uteri being <26 mg in Ovx mice (Fig. 1*B*). The duration of E_2_ deficiency did not affect the uterine mass (*P* = 0.393). Mass of TA muscles was 6% greater at 14 compared with 6 days post-Ovx (*P* = 0.040) but did not differ between Sham and Ovx (*P* = 0.471; Fig. 1*C*).

### Effects of Ovarian Hormones and ERα Signaling on Satellite Cell Number

To identify the best time post-Ovx to study satellite cell cycling, FACS was used to quantify the total number of satellite cells (lineage negative; VCAM α7 double-positive cells) in TA and GC muscles (Supplemental Fig. S1, *A* and *B*; see https://doi.org/10.6084/m9.figshare.17096900.v1 respectively). An interaction between group and time was observed for satellite cell number in the TA (*P* = 0.049; Fig. 1*D*). Satellite cell number did not differ between Sham and Ovx at 6 or 10 days postsurgery (*P* ≥ 0.862); however, was 33% lower in Ovx than Sham mice at 14 days (3,738 ± 128 vs. 5,554 ± 354, respectively; *P* = 0.010; Fig. 1*D*). We evaluated the density of satellite cells, calculated by dividing the absolute cell number by the wet mass of each muscle. Satellite cell density in TA muscles was lower at 14 days compared with 10 days (time effect; *P* = 0.031; Fig. 1*E*). Similarly, satellite cell number and density were 33% lower in GC muscles from Ovx than Sham mice at 14 days postsurgery (group effect; *P* ≤ 0.001; Supplemental Fig. S1, *C*–*E*). Compilation of satellite cell number at these early time points of ovarian hormone deficiency with later time points previously reported (i.e., 56, 112, and 196 days post-Ovx; 58), identifies 14 days as the earliest time point analyzed where a decline in satellite cell number is measured (Supplemental Fig. S1, *F* and *G*). Pax7-immunostaining of TA muscle cross sections at 14 days post-Ovx showed 30% and 32% fewer satellite cells per cross section and per mm^2^, respectively (*P* ≤ 0.025; Fig. 1, *F* and *G*), recapitulating the decline in satellite cell number at 14 days post-Ovx observed with FACS quantification.

**Figure 2.**
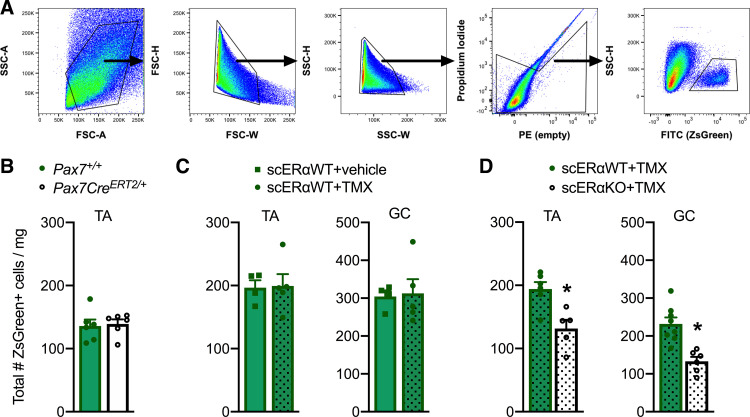
Satellite cell number with the loss of E_2_-ERα signaling. *A*: gating scheme for ZsGreen+ cells in tibialis anterior and gastrocnemius muscles. ZsGreen+ cells were gated based on forward/side scatter (*plots 1–3*) and live cells (propidium iodide negative – *plot 4*). These cells were then selected for ZsGreen-positive cells SSC-H X FITC (ZsGreen; absolute). Total number of ZsGreen+ satellite cells normalized to muscle masses from *Pax7^+/+^* (scERαWT; *n* = 6) and *Pax7^CreERT2/+^* (scERαKO; *n* = 6) female mice *(B)*, scERαWT female mice treated with vehicle (*n* = 4) or TMX (*n* = 5) *(C)*, and scERαWT and scERαKO female mice treated with TMX (*n* = 5–8/group; *D*). For *C* and *D*, TA and GC muscles were harvested and analyzed 14 days after treatment or the loss of E_2_-ERα signaling. Values are presented as means ±SE. *Different from scERαWT. ERα; estrogen receptor α; E_2_; estradiol; GC, gastrocnemius; TA; tibialis anterior; TMX; tamoxifen.

We recently determined that *Esr1*, the gene encoding estrogen receptor α (ERα), is more highly expressed in satellite cells than the two other estrogen receptors, *Esr2* (ERβ) and *Gper*, and the progesterone receptor, *Pgr* (60). This led us to develop an inducible satellite cell-specific ERα knockout mouse (scERαKO) to specifically probe E_2_-ERα signaling in satellite cells (described in Ref. 60) by measuring ZsGreen+ cells (Fig. 2*A*). First, we completed control experiments to directly show that the presence of *Pax7^CreERT2/+^* and tamoxifen treatment did not influence satellite cell numbers (Fig. 2, *B* and *C*). Next, we measured ZsGreen+ cells in TA and GC muscles from scERαKO and control littermates (scERαWT) 14 days after ablation of ERα. Similar to Ovx mice, scERαKO mice have 24%–62% fewer satellite cells (*P* ≤ 0.050; Fig. 2*D*) indicating that E_2_ deficiency drives the loss of satellite cells with Ovx as opposed to any other ovarian hormone. Accuracy of satellite cell counts by FACS was confirmed by the concurrent analysis of flow cytometry counting beads and ZsGreen+ satellite cells (Supplemental Fig. S2, *A* and *B*; see https://doi.org/10.6084/m9.figshare.17096915.v1). Together, these results indicate that deficiency of the hormone E_2_ drives the loss of satellite cells with Ovx as opposed to any other ovarian hormone and that the loss of E_2_ or its receptor for only 14 days causes a reduction in the number of satellite cells in skeletal muscles of female mice. Importantly, these data identify the most appropriate time point for conducting the following in vivo experiments to investigate impaired satellite cell cycling as a mechanism for the decline in satellite cell number with disruption of E_2_-ERα signaling.

### Effects of E_2_ on Satellite Cell Cycle Progression

**Figure 3.**
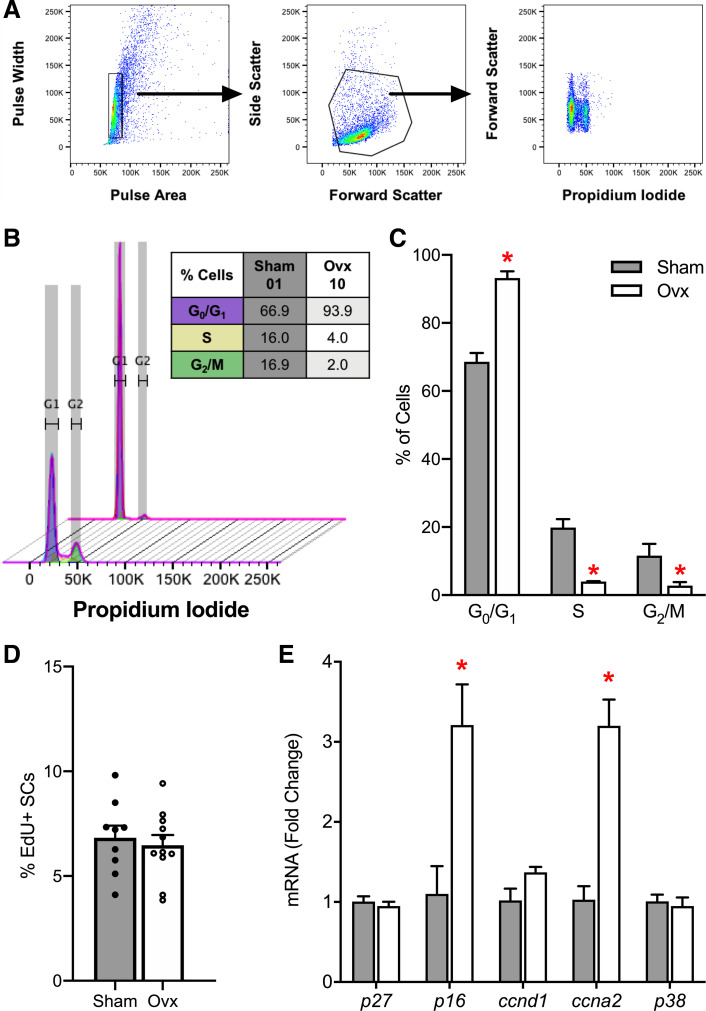
Role of E_2_ in satellite cell cycle progression. *A*: cell cycle analysis by quantitation of DNA content via flow cytometry. Sorted satellite cells were fixed and gated based on pulse area/width (*plot 1*), forward/side scatter (*plot 2*), and propidium iodide histogram plot (*plot 3*). *B*: overlay histogram of the cell cycle analysis and table of cell cycle distribution of satellite cells from representative Sham (*sample 01*) and Ovx (*sample 10*) TA muscles. *C*: cell cycle distributions for satellite cells in G_1_, S, and G_2_ phases (*n* = 4/group). *D*: in vivo percentage of EdU+ satellite cells 21 days post-Ovx (*n* = 9–11/group). *E*: RT-qPCR mRNA expression of cell cycle-related genes in ZsGreen+ satellite cells isolated from hindlimb muscles of Sham (*n* = 4) and Ovx mice (*n* = 4) at 14 days postsurgery. Values are presented as means ± SE. *Different from Sham. E_2_; estradiol; Ovx; ovariectomized; SC; satellite cell; TA; tibialis anterior.

First, we investigated whether the decline in satellite cell number with E_2_-ERα disruption is due to changes in satellite cell cycle progression. The most common method for evaluating the cell cycle is DNA content; thus, we used isolated satellite cells from Sham and Ovx mice 14 days postsurgery and stained the DNA stoichiometrically with PI (Fig. 3*A*). This analysis revealed significant differences between Sham and Ovx mouse muscles in the distribution of satellite cells in each cell cycle phase (*P* ≤ 0.048; Fig. 3, *B* and *C*). To evaluate the percentage of S-phase satellite cells long-term, Sham and Ovx mice received EdU slow-release pellets, implanted on the day of Ovx surgery, for 21 days (Supplemental Fig. S3*A*). Flow cytometry analysis indicated that the percentage of EdU+ satellite cells accumulated over 21 days did not differ between TA muscles from Sham and Ovx mice (*P* = 0.646; Fig. 3*D*). qPCR analysis showed that gene expression of *p16^INK4a^*, a negative regulator of the cell cycle, and *ccna2*, a regulator of both DNA replication and mitotic entry, were upregulated threefold in satellite cells from Ovx mice (*P* ≤ 0.020; Fig. 3*E*).

### Effects of E_2_ on Proliferation and Cell Cycle Kinetics of Satellite Cells In Vitro

**Figure 4.**
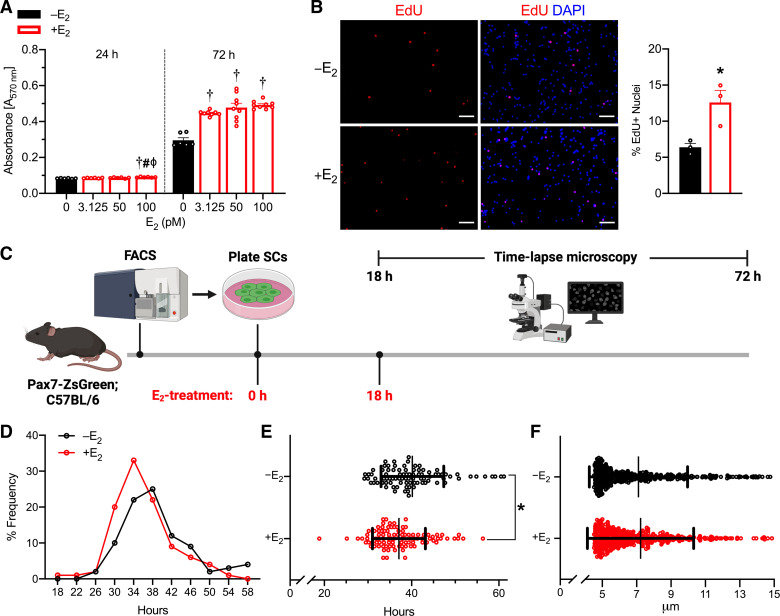
Proliferation and cell cycle kinetics of E_2_-treated satellite cells. *A*: Formazan absorbance expressed as a measure of cell viability from satellite cells treated with physiological doses of E_2_ (*n* = 6–9/group). *B*: representative images and quantification of the percentage of EdU+ nuclei 6 days postplating of satellite cells (*n* = 3/group). Scale bars = 50 µm. *C*: schematic of study design for time-lapse microscopy experiment to quantify satellite cell time to first division. *D*: frequency of satellite cell first division (*n* = 3/group). *E*: mean time of satellite cell first division. *F*: satellite cell size at 18 h after plating and initial E_2_-treatment. E_2_; estradiol. Values are presented as means ± SE in *A* and *B* and means ± SD for *E* and *F*. †Different from 0 (vehicle), #different from 3.125 pM, ϕdifferent from 50 pM at corresponding time points. *Different from –E_2_. E_2_; estradiol.

To further characterize the impaired cycling of the satellite cell pool with E_2_-ERα disruption, we isolated satellite cells from female Pax7-ZsGreen mice and assessed cell cycle kinetics in vitro. First, we evaluated satellite cell proliferation at 24 and 72 h postplating with E_2_ (final concentrations 0, 3.125, 50, and 100 pM). Satellite cell proliferation was 51%–67% greater with E_2_-treatment at 72 h postplating, regardless of the dose (*P* < 0.001; Fig. 4*A*). The 100 pM E_2_ concentration was used for all subsequent experiments, as it significantly affected satellite cell proliferation at both 24 and 72 h postplating and represents physiologically relevant E_2_ levels in mice (66). Furthermore, satellite cell proliferation measured by ATP luciferase assay was six- and sevenfold greater with 100 pM E_2_ treatment at 4 and 6 days after plating, respectively (*P* < 0.001; Supplemental Fig. S4; see https://doi.org/10.6084/m9.figshare.17096927.v1), and the percentage of EdU+ nuclei was twofold greater with 100 pM E_2_ treatment at 6 days after plating (*P* = 0.025; Fig. 4*B*).

To measure the rate of satellite cell division, we treated satellite cells with E_2_ at 0 and 18 h after plating and observed cell division by time-lapse imaging from 18 to 72 h after plating (Fig. 4*C*). We found that E_2_-treated satellite cells require less time to complete the first division compared with vehicle-treated (37.1 ± 0.6 and 40.2 ± 0.8 h; *P* = 0.001; Fig. 4, *D* and *E*). Since cell size is proposed to be indicative of cell growth, we measured satellite cell size at 18 h postplating; E_2_ treatment did not affect satellite cell size (*P* = 0.547; Fig. 4*F*).

### Effects of E_2_ on Satellite Cell Colony-Forming Ability and Differentiation

**Figure 5.**
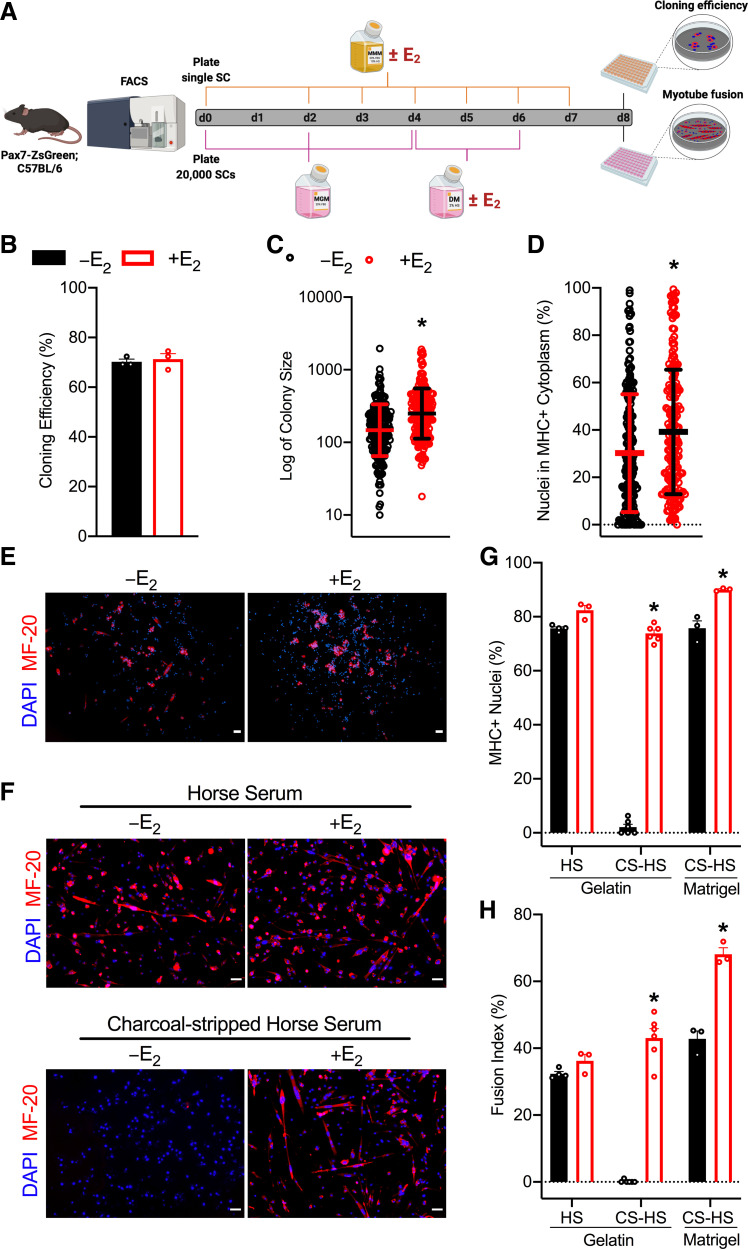
Effects of E_2_ on satellite cell colony-forming ability and differentiation. *A:* schematic for colony-forming assay (*top*) where single satellite cells were plated and treated with MMM with vehicle or E_2_ until *day 8* when colony number, size, and spontaneous differentiation are measured (*n* = 3/group). Schematic for differentiation assay (*bottom*) where 20,000 satellite cells were plated and treated with vehicle or E_2_ only after switching to low-serum differentiation medium (*n* = 3–6/group). *B*: clonal efficiency quantified by counting the number of colonies in each plate and the number of wells in which a single cell was sorted. *C*: geometric mean of colony size. *D*: percentage of nuclei in MHC+ cytoplasm. Representative images of immunofluorescence of MF-20 (MHC) and DAPI on a gelatin matrix after 8 days of MMM with or without E_2_ *(E)* or after low-serum medium conditions with normal horse serum (HS; *top*) and charcoal-stripped horse serum (CS-HS; *bottom*) for 3.5 days with or without E_2_ *(F)*. Scale bars = 50 µm. *G*: percentage of MHC+ nuclei. *H:* quantitative analysis of myotube fusion index. Values are presented as means ± SE for *B*, *D*, *G,* and *H* and means ± SD for *C*. *Different from –E_2_ within condition. CS-HS; charcoal stripped horse serum; DM; differentiation medium; E_2_; estradiol; FACS; fluorescence-activated cell sorting; HS; horse serum; MGM; muscle growth medium; MHC; myosin-heavy chain; MMM; mouse myoblast medium; SC; satellite cell.

To assess clonogenicity of single satellite cells with and without E_2_, we treated single satellite cells with vehicle or E_2_ and allowed colonies to form for 8 days (Fig. 5*A,* *top*). The ability of the cells to survive and form colonies in vitro was not affected by E_2_ treatment (*P* = 0.687; Fig. 5*B*). However, mean colony size and spontaneous differentiation, quantified as nuclei in myosin heavy chain (MHC)+ cytoplasm, were ∼69% and 30% greater with E_2_ treatment (*P* < 0.001; Fig. 5, *C* and *D*, respectively). Because we observed greater satellite cell proliferation in vitro with E_2_ treatment under standard culture conditions (Fig. 4, *A* and *B*; Supplemental Fig. S4), to evaluate the effects of E_2_ on satellite cell differentiation alone, we cultured satellite cells under identical conditions until confluent and then began vehicle or E_2_ treatment after switching to low-serum differentiation medium (Fig. 5*A,* *bottom*). The number of nuclei in MHC+ myotubes and fusion index did not differ between –E_2_ and +E_2_ groups when normal HS was used (*P* = 0.053 and *P* = 0.180, respectively; Fig. 5, *F–H*). However, the number of nuclei in MHC+ myotubes and fusion index was greater with E_2_ treatment compared with no E_2_ when charcoal-stripped serum was used regardless of the matrix (*P* ≤ 0.033 and *P* ≤ 0.001, respectively; Fig. 5, *F–H* *middle* and *right* bars). In fact, myotubes were almost nonexistent in the –E_2_, vehicle-treated wells on a gelatin matrix, supporting the concept that the lack of E_2_ impairs terminal differentiation (Fig. 5*F*; −E_2_ on *left*).

## DISCUSSION

Recent developments in satellite cell biology have highlighted the importance of circulating factors, such as sex hormones, in skeletal muscle growth and regeneration. Here, we expanded upon our previous work demonstrating that there is a substantial decline in the number of satellite cells in muscles from female mice with an ovarian hormone deficiency, which can be prevented with E_2_ treatment (60). Results of the present study show that deficiency of the hormone E_2_ drives the loss of satellite cells with Ovx as opposed to any other ovarian hormone and that the loss of E_2_ or its receptor for only 14 days impairs satellite cell maintenance. We show mechanistically that impaired satellite cell maintenance caused by E_2_ deficiency includes altered satellite cell cycle progression, kinetics, proliferation, and differentiation.

When satellite cells exit quiescence, the noncycling G_0_ phase, they can adopt different cell fates: differentiation, cell death (i.e., apoptosis, necrosis, and autophagy), or senescence. These satellite cell fate decisions are carefully regulated by intrinsic and extrinsic cues and significant alterations can lead to exhaustion of the satellite cell pool (14, 69, 70). In vivo FACS analysis of cell cycle distribution at 14 days post-Ovx indicated that E_2_-deficient satellite cells have impaired cell cycle progression at both G_1_ to S and S to G_2_ transitions. There were four- to fivefold fewer satellite cells from Ovx than Sham muscles in S-phase and G_2_-phase (Fig. 3*C*). In contrast, 93% of the satellite cell population was in G_0_ phase in Ovx muscles versus only 69% for Sham (Fig. 3*C*). Theoretically, there is no need for elevated cell cycling or proliferation in the ovary-intact mice because the satellite cell pool is maintained. However, given that there are ∼33% fewer satellite cells in muscles of Ovx mice compared with ovary-intact mice, we proposed that there would be an increase in proliferating satellite cells to counter the loss of satellite cells caused by E_2_ deficiency previously suggested to occur through apoptosis (60). We found that the cumulative proportion of proliferating satellite cells in vivo over 21 days is the same in muscles of Ovx and ovary-intact mice (Fig. 3*D*) and that the percentage of EdU-labeled satellite cells was similar to that previously reported. Specifically, studies have shown that 1%–2% of satellite cells are labeled per week (71) or 0.2 ± 0.1% of satellite cells are labeled per day (72), suggesting that after 21 days ∼6% of satellite cells will be EdU+, which is supported by our observations in this study. Interestingly, in vitro, we observed that E_2_-treatment of isolated satellite cells have 97% greater EdU incorporation compared with vehicle-treated satellite cells (Fig. 4*B*). These results are consistent with other reports showing that disruption of E_2_-ERα/β signaling impairs the proliferation of cultured satellite cells or exercise-induced satellite cell proliferation (53–56, 73, 74).

Consequently, we evaluated genes that are known to regulate satellite cell cycle progression. The progression of satellite cells from G_1_ to S phase is promoted by insulin-like growth factor-1 (IGF-1) via downregulation of *p27^Kip1^* (75) and *ccnd1* via downregulation of transforming growth factor β (TGF-β) signaling (76), whereas accumulation of cyclin-dependent kinase (CDK) inhibitors, such as *p16^INK4a^*, prevents satellite cell G_1_ to S phase progression resulting in cell cycle arrest and replicative senescence (14). Changes in satellite cell cycling in a short period of time are also a result of rapid molecular changes via protein-kinase cascades, such as phosphatidylinositol 3-kinase (PI3K; 52, 55). E_2_ deficiency did not affect *p27^Kip1^* or *ccnd1* mRNA expression (Fig. 3*E*), supporting the notion that *p27^Kip1^*, a negative regulator of cell cycling, is not associated with E_2_-dependent satellite cell proliferation, and potentially occurs through PI3K as previously demonstrated (52, 55). We instead show a threefold upregulation in mRNA expression of *p16^INK4a^*, a marker of permanent cell cycle arrest, in satellite cells from muscles of Ovx mice (Fig. 3*E*). Sousa-Victor et al. (14) recently showed for the first time that the loss of cell cycle protective mechanisms with age results in senescent *p16^INK4a^*-expressing satellite cells. That study revealed a two- and fourfold upregulation in *p16^INK4a^* mRNA expression and 15% and 40% SA-β gal+ satellite cells from 28 to 32 mo geriatric mice and 75 yr humans compared with young adults, respectively (14). With *p16^INK4a^*-induced replicative senescence playing a role in satellite cell maintenance in aged mice and humans, it will be interesting to further investigate whether the inability of satellite cells to efficiently cycle and generate progeny in an environment lacking E_2_ is due to *p16^INK4a^*-induced replicative senescence and whether the loss of E_2_ stimulates a switch to a senescent, nonproliferative state or impairs the maintenance of quiescence.

We also present data that mRNA expression of *ccna2*, an essential regulator of both the onset of the S-phase transition and during the G_2_-M transition, is upregulated (Fig. 3*E*). This result is in contrast to a previous study that shows a decline in *ccna2* mRNA expression at 6 days postinactivation of ERβ signaling in satellite cells (73). The discrepancy between these studies is presumably due to different analysis time points, hormone versus receptor signaling, and that the present study did not culture satellite cells before qPCR analysis as was done previously (72).

It may seem paradoxical that both *p16^INK4a^* and *ccna2* are upregulated in satellite cells from Ovx muscles, but it is important to note that our study involved a satellite cell population analysis and not an individual satellite cell-based analysis. As such, we do not know if the *p16^INK4a^*-expressing satellite cells were the same or different satellite cells expressing *ccna2*. Functionally heterogeneous subpopulations of satellite cells have been previously identified using techniques such as single-cell RNA-sequencing (77–79), single-cell mass cytometry (CyTOF; 80), lineage tracing (7, 8), and label retaining (81, 82). These cell cycle gene expression data along with our finding that E_2_ is necessary for transitions between cell cycle phases strongly suggest that reduced cell cycle progression is one of the mechanisms whereby satellite cell number declines with E_2_ deficiency.

Satellite cell subpopulations are distinguished by differential expression of genes or cell surface markers, or phenotypic changes (e.g., time to first division). For instance, noncycling satellite cells, in G_0_ phase, express high levels of *Sprouty1* and *p27^Kip1^* to maintain quiescence (77, 81, 83) and can reversibly transition from G_0_ to a primed G_Alert_ phase permitting rapid cell cycle entry (7). We therefore cultured satellite cells in the presence or absence of E_2_ and observed time to first division. To note, normal serum can contain estrogens from female donors (84), so we utilized serum where the endogenous estrogens were removed by charcoal-stripping and then controlled estrogen exposure by adding specific amounts of exogenous E_2_. We observed that E_2_-treated satellite cells have reduced time to the first division, only taking 33–48 h to undergo the first division (Fig. 4*E*). The time to first division was not comparable to that of satellite cells from muscles 3 days postinjury, which is reported to be less than 20 h (7). Other time-lapse microscopy studies have shown that 16% of satellite cells do not divide and it takes the remaining satellite cells 36–48 h to undergo cell division (7, 85). It is worth mentioning that satellite cells grow more slowly in a medium containing charcoal-stripped serum than in normal serum, so the time to first division observed of vehicle-treated satellite cells in our study may not closely compare with previous satellite cell studies. Advancements in cell-labeling techniques have allowed analysis of satellite cell division history revealing that satellite cell division can be separated into slow- and fast-dividing subpopulations. Studies have shown that the slow-dividing subpopulation accounts for 10%–20% of satellite cells and are the long-term self-renewing population; whereas the fast-dividing subpopulation accounts for 80%–90% of satellite cells, which generate a great deal of differentiated cells but have limited replication (86–89). This study showed a faster rate of cell division with E_2_ treatment compared with vehicle treatment, suggesting that the satellite cells exhausted in an environment without E_2_ are from the fast-dividing subpopulation. These results identify impaired cell cycle kinetics as an additional mechanism, whereby the absence of E_2_ influences satellite cell maintenance.

Our study used a variety of methods to analyze the satellite cell proliferation in environments with and without E_2_, including colorimetric assays that measure metabolic activity (i.e., MTT, ATP luciferase), fluorescent dyes (i.e., PI), and incorporation of thymidine analogs (i.e., EdU). The collective data suggest that satellite cell viability and proliferation are enhanced when E_2_ is present (Fig. 4, *A* and *B*; Supplemental Fig. S4). Previous rodent studies investigating the effects of E_2_ on satellite cell proliferation did not observe changes in satellite cell proliferation with E_2_-treatment under normal conditions (i.e., without injury or exercise), when quantifying proliferation using the proliferation marker, proliferating cell nuclear antigen (PCNA; 54) or immunostaining for incorporated thymidine analog BrdU (53). Kamanga-Sollo et al. also used radioactive thymidine (^3^H-thymidine) in vitro and demonstrated greater proliferation in bovine satellite cells treated with E_2_ when medium is free of IGF binding protein (IGFBP)-3, a protein previously shown to antagonize IGF-1 actions on myogenic proliferation (90). The 1.5-fold increase in bovine satellite cell proliferation noted in their study was only observed when bovine satellite cells were cultured with 10^4^ pM E_2_ and not 10^3^ pM E_2_ (52, 74). Considering that circulating E_2_ concentrations in rodent models and premenopausal women range from ∼5 to 200 pM (91, 92), all of the currently published studies evaluating the effects of E_2_ on cultured satellite cells used supraphysiological E_2_ doses [ranging from 10^4^ to 10^6^ pM E_2_; (52, 58, 74, 93)]. Higher doses of E_2_ were possibly used due to the short half-life of E_2_ in culture (presumably 3 h), relatively high photodegradation half-life in solution (94), and adherence of E_2_ molecules to polypropylene (95). Here, we use physiologically relevant E_2_ doses to establish a final concentration of 100 pM E_2_ in culture and treated either every 24 or 48 h. Our data demonstrated greater proliferative capacity of satellite cells with E_2_ compared with vehicle at 3.125, 50, and 100 pM (final concentrations) at 72 h postplating (Fig. 3*A*). These findings suggest that previous studies using supraphysiological E_2_ doses may have observed opposite effects compared with those observed with physiological ranges, as has been shown in other cells/tissues (96, 97).

Assessment of the colony-forming ability of single satellite cells demonstrated that all satellite cells were able to generate clones, regardless of treatment, but the satellite cells supplemented with E_2_ had greater colony sizes, suggesting enhanced proliferative capacity (Fig. 5*C*). Satellite cell-derived myoblasts treated with E_2_ had augmented differentiation, indicated by increased MHC+ nuclei and myotube fusion index (Fig. 5, *G* and *H*). Other studies have yielded similar results using other methodologies. Kitajima et al. (98) cultured myofibers from Ovx in floating conditions for 3 days and observed significantly lower numbers of differentiating satellite cells compared with those from ovary-intact mice, and Galluzzo et al. (59) found that E_2_-treated rat myoblast cells (L6) had augmented expression of differentiation markers, MHC and myogenin. On the contrary, Ogawa et al. (58) previously demonstrated that satellite cells treated with E_2_ (10^4^ pM) and cultured for 8 days in differentiation medium displayed inhibited myogenesis and reduced fusion index . We propose this negative regulation by E_2_ is due to supraphysiological dosing and the prolonged duration of culture in the differentiation medium.

The present study showed no noticeable difference in myotube fusion index when satellite cells were cultured in normal HS (Fig. 5*H*). This result emphasizes the importance of using charcoal-stripped serum to deplete E_2_ and other endogenous nonpolar lipid-bound materials (e.g., hormones, growth factors, and cytokines), which have demonstrated estrogenic activity and could potentially confound results (99, 100). Given that the pH indicator phenol-red has also been shown to have estrogenic effects (101–103), this study used phenol-red free DMEM with CS-HS. The absence of myotubes in vehicle-treated satellite cells cultured in phenol-red free DMEM with CS-HS on a gelatin matrix (Fig. 5*F*) demonstrates that E_2_, possibly as well as the other lipid-modified proteins, are crucial for myoblast differentiation. In addition, repeating these conditions with a Matrigel matrix indicates that E_2_ is largely beneficial at the terminal differentiation stage (i.e., fusion of myocytes to form myotubes; Fig. 5*F*). These results suggest that E_2_ regulates both cell division of undifferentiated satellite cells and fusion of differentiated myocytes, resulting in enhanced myogenesis.

In summary, we show that mechanisms underlying the E_2_ deficiency-induced decline in satellite cell number are multifactorial, involving impaired satellite cell cycle progression, kinetics, proliferation, and differentiation. These findings have implications in the preservation of efficient muscle regeneration, including targeting p16-mediated pathways to prevent cell cycle arrest of satellite cells and subsequent exhaustion of the satellite cell pool and are relevant to all women experiencing a decline in circulating E_2_ levels. Ultimately, understanding how E_2_ regulates satellite cells will help to determine therapies for improving muscle regeneration and recovery of strength that affect the quality of life.

## DATA AVAILABILITY

The data that support the findings of this study are available from the corresponding author upon reasonable request.

## SUPPLEMENTAL DATA

10.6084/m9.figshare.17096900.v1 Supplemental Fig. S1: https://doi.org/10.6084/m9.figshare.17096900.v1.

10.6084/m9.figshare.17096915.v1 Supplemental Fig. S2: https://doi.org/10.6084/m9.figshare.17096915.v1.

10.6084/m9.figshare.17096918.v1 Supplemental Fig. S3: https://doi.org/10.6084/m9.figshare.17096918.v1.

10.6084/m9.figshare.17096927.v1 Supplemental Fig. S4: https://doi.org/10.6084/m9.figshare.17096927.v1.

## GRANTS

This study was funded by the National Institute of Health (NIH) Grants R01-AG062899 (D.A.L. and M.K.) and R01-AG031743 (D.A.L.). A.A.L. was supported on T32-AG029796 and S.L.M. was supported on T32-AR007612.

## DISCLOSURES

No conflicts of interest, financial or otherwise, are declared by the authors.

## AUTHOR CONTRIBUTIONS

A.A.L., S.L.M., E.B., M.K., and D.A.L. conceived and designed research; A.A.L., A.S.S., S.L.M., B.P.S., C.V., and E.B. performed experiments; A.A.L., A.S.S., B.P.S., and Z.A.R. analyzed data; A.A.L., S.L.M., M.K., and D.A.L. interpreted results of experiments; A.A.L., A.S.S., and B.P.S. prepared figures; A.A.L. drafted manuscript; A.A.L., S.L.M., E.B., M.K., and D.A.L. edited and revised manuscript; A.A.L., A.S.S., S.L.M., B.P.S., C.V., Z.A.R., E.B., M.K., and D.A.L. approved final version of manuscript.
